# CONECT: Novel Weighted Networks Framework Leveraging Angle-Relation Connection (ARC) and Metaheuristic Algorithms for EEG-Based Dementia Classification

**DOI:** 10.3390/s25247439

**Published:** 2025-12-07

**Authors:** Akashdeep Singh, Supriya Supriya, Siuly Siuly, Hua Wang

**Affiliations:** 1 Institute for Sustainable Industries and Liveable Cities, Victoria University, Melbourne, VIC 3011, Australia; siuly.siuly@vu.edu.au (S.S.); hua.wang@vu.edu.au (H.W.); 2 Centre for Artificial Intelligence Research and Optimisation (AIRO), Torrens University, Melbourne, VIC 3000, Australia; supriya.supriya@torrens.edu.au

**Keywords:** Alzheimer’s disease, brain networks, CONECT, ARC rule, electroencephalography, feature engineering, frontotemporal dementia, machine learning, geometric connection, complex networks

## Abstract

Accurate and robust classification of dementia subtypes using non-invasive electroencephalography (EEG) signals remains a critical challenge for clinicians and researchers in the field of neuroscience. Traditional methods often rely on limited spectral features, overlooking the rich structural and geometric information inherent in EEG dynamics. CONECT (Complex Network Conversion and Topology), a novel framework, is introduced and built upon four core innovations. First, EEG time series are transformed into weighted networks using a novel Angle-Relation Connection (ARC) rule, a geometry-based approach that links time points based on angular monotonicity. Secondly, a tunable edge-weighting function is introduced by integrating amplitude, temporal, and angular components, providing adaptable heuristics adaptable to the most promising biomarker, i.e., curvature-driven features in dementia. Additionally, two new graph-based EEG features, the Weighted Angular Irregularity Index (WAII) and the Curvature-Based Edge Feature Index (CBEFI), are proposed as potential biomarkers to capture localized irregularity and signal geometry, respectively. For the first time in a dementia EEG classification study using the OpenNeuro ds004504 dataset (raw), Ant Colony Optimization (ACO) is applied as a feature selection technique to select the most discriminative features and improve model classification and transparency. The classification results demonstrate CONECT’s potential as a promising, interpretable, and geometry-informed framework for accurate and practical dementia subtype diagnosis.

## 1. Introduction

Dementia is a progressive neurodegenerative condition, becoming worse over time because it is caused by the gradual loss of neurons and brain tissue (degeneration). It is becoming a major global health crisis around the world [1]. More than 57 million people had dementia in 2021 [2]. It is predicted that this number would grow to 78 million by 2030 and 139 million by 2050 [3]. Alzheimer’s disease (AD) is the most prevalent type of dementia. Frontotemporal dementia (FTD) is the second most frequent type, and it affects younger people more than older ones. Despite increasing awareness, around 75% of cases around the world remain undiagnosed globally, resulting in missed opportunities for timely intervention and appropriate care [4]. This means that people do not obtain the help they need when they need it. The differentiation between subtypes such as Alzheimer’s Disease (AD) and Frontotemporal Dementia (FTD) remains a diagnostic problem due to their overlapping cognitive and behavioral manifestations [5,6]. A lot of people receive the wrong diagnosis (misclassification), which makes treatment less effective and makes it harder for patients and their families to receive aid. Dementia is on the rise, which is putting a lot of burden on caregivers, healthcare systems, and society as a whole. Therefore, it is very crucial to diagnose dementia early and correctly to ensure that focused therapies are given, patient outcomes are improved, and the worldwide economic burden of dementia is lowered.

Electroencephalography (EEG) is considered the most desirable modality for dementia diagnosis because it is non-invasive, reasonably priced, broadly deployable, and sensitive to abnormalities in neural dynamics of the brain [7,8]. In comparison to structural neuroimaging and invasive biomarkers (intracranial EEG (iEEG) or electrocorticography (ECoG)), EEG facilitates frequent and clinically friendly monitoring of brain functional activity. Traditional EEG pipelines for monitoring the brain activities are usually centered on spectral measures such as an increase in the delta or theta rhythm, reductions in the alpha and beta rhythms of the brain EEG, pairwise coherence, and entropy-based complexity indices [9,10,11]. These statistical features were able to capture the broad variations in oscillatory activities and synchronization in Alzheimer’s disease (AD), and played a significant role in several successful binary classification tasks that distinguish AD from Healthy Controls (HC) [12,13,14,15]. However, they have three strong limitations: (i) Insufficient sensitivity to subtle, nonlinear pattern, and topological (brain connection) disruptions preceding overt decline; (ii) The focus of the classification problem is narrow, with limited attention to multiclass classification that comprises frontotemporal dementia (FTD), and (iii) The interpretability is also limited because most of the high-performing models operate as black box decision systems with limited clinical transparency.

Current research has shifted the paradigm of EEG signal analysis from isolated signal descriptors toward complex network–based models where EEG activity is mapped to graphs in which EEG channels or data points are represented by nodes and edges encode connectivity or similarity [16,17,18,19]. To quantify and understand brain dynamics, the network analyst has developed a principled vocabulary of topological features (such as clustering coefficient, path length, global efficiency, modularity, and small-world topology). These features have produced significant results when deployed on EEG dementia classification problems. For example, reduced global integration, fragmentation of long-range connectivity, and deviations from small-world topology in AD. Such results confirm that network structure carries discriminative information beyond univariate spectra [19,20,21].

However, classical graph-based techniques have critical constraints. Edge weights are generally discarded in threshold or binary adjacency matrices, thereby compressing the rich variability in connection strength. Conventional techniques of time-series-to-network mapping (i.e., Visibility Graphs (VGs) and variants) define the edges between the nodes based on vertical line-of-sight comparisons only [22,23,24]. While effective for some nonlinear signatures, these mappings do not encode trajectory geometry (directionality, angular monotonicity) or curvature of the evolving signal. As a result, EEG signal-based networks may miss fine-grained waveform perturbations that are important characteristics of neurodegeneration. Additionally, as feature sets expanded, many studies relied on manual selection of the features or ad hoc filtering, resulting in selection bias and instability. Similarly, applying complex classifiers (e.g., deep ensembles, etc.) improved classification performance metrics but degraded interpretability and attribution fidelity, thus limiting clinical transparency [13,25,26,27,28].

To address these gaps, we introduce a geometry-aware network framework named CONECT (Complex Network Conversion and Topology). First, the EEG signals are mapped to a complex network based on the novel Angle-Relation Connection (ARC) rule. Then the weight of the edges is determined using a new Adaptive edge weighting equation. Each ARC edge is assigned a weight combining amplitude (α) difference, temporal (β) separation, and angular (γ) orientation, modulated by tunable parameters (α, β, γ). Once weighed, the CONECT network is constructed and analyzed using a comprehensive set of 15 features (such as clustering coefficient, degree distribution, and global efficiency), as well as two novel indices: the Weighted Angular Irregularity Index (WAII) and the Curvature-Based Edge Feature Index (CBEFI). To the best of our knowledge, this is the first application of ACO to EEG-based dementia classification, and specifically the first deployment on OpenNeuro ds004504 (AD, FTD, and healthy controls). ACO reduces dimensionality, improves generalization, and enhances interpretability by repeatedly surfacing the most informative features. The classification performance of the CONECT framework is evaluated on an online benchmark dataset, i.e., OpenNeuro ds004504, using five machine learning classifiers: Random Forest, AdaBoost, Bagged Trees, k-Nearest Neighbors (k-NN), and Radial Basis Function Support Vector Machine (RBF-SVM). The Proposed framework achieves high accuracy (88.02%) for multiclass (AD vs. FTD vs. HC) classification and 97.5% accuracy in FTD vs. HC.

The key novelties and major contributions of this study are as follows:

1. *Geometry-aware weighted graph construction for EEG:* We introduce the ARC rule, based on a directed, angular-monotonicity criterion that integrates signal direction and curvature into connection. This advances the visibility-style mappings that are based on amplitude comparisons.
2. *Adaptive, multi-factor edge weighting:* We propose a tunable edge weighting equation (α, β, γ) that combines amplitude difference, temporal distance, and angular orientation for better application (disease problem) of adaptive and information-dense networks.
3. *Two novel features as biomarkers (WAII and CBEFI):* We developed two graph-based novel features that capture local irregularity and global curvature, providing additional geometric information absent from traditional graph metrics.
4. *First use of ACO for dementia EEG feature selection:* First-time deployment of ACO on OpenNeuro ds004504 to discover crucial and discriminative feature subsets, enhancing performance and model interpretability.
5. *Raw-EEG benchmarking:* The proposed framework is tested on unprocessed EEG recordings (OpenNeuro ds004504) without ICA or manual artifact removal, demonstrating robust performance under realistic signal conditions.

The remainder of the paper is structured as follows. Section 2 details the CONECT methodology comprising the dataset used, ARC construction, adaptive weighting, the 15-feature graph features including WAII/CBEFI, and ACO-guided selection, followed by classification and performance metrics used. Section 3 reports results and discussion on OpenNeuro ds004504, including multiclass and binary performance, feature-importance analyses, implications for clinical deployment, and interpretability.

## 2. Materials and Methods

This section provides in-depth detail about the proposed CONECT (Complex Network Conversion and Transformation) framework. It covers the importance of the novel Angle-Relation Connection (ARC) Rule in the EEG signals for complex network transformation. In the feature extraction section, two newly developed features (Weighted Angular Irregularity Index (WAII) and the Curvature-Based Edge Feature Index (CBEFI) are proposed that provide valuable insight about topological complexity and temporal geometry of EEG signals. In the feature selection section, we discussed the rationale for the first-time use of the Ant Colony Optimization algorithm in feature selection for dementia-related EEG signal analysis. Overall, the CONECT framework comprises five key stages:

1. *Dataset Description:* An overview of the online benchmark EEG dataset (i.e., OpenNeuro ds004504 (https://openneuro.org/datasets/ds004504/versions/1.0.8?utm_source (accessed on 15 October 2025))), used in this study.
2. *Network Construction:* Transformation of EEG signals into complex networks via the novel Angle-Relation Connection (ARC) Rule, with tunable heuristic edge weight values.
3. *Feature Extraction:* To extract the valuable or meaningful pattern from the above-constructed weighted network, a total of 15 features were extracted, including two that are newly proposed (WAII and CBEFI).
4. *Feature Selection:* ACO is deployed as a feature selection method for identifying the most discriminative feature subsets among 15 features through a bio-inspired search strategy.
5. *Classification*: The performance of the CONECT framework is evaluated using five different machine learning classifiers.

**Figure 1:**
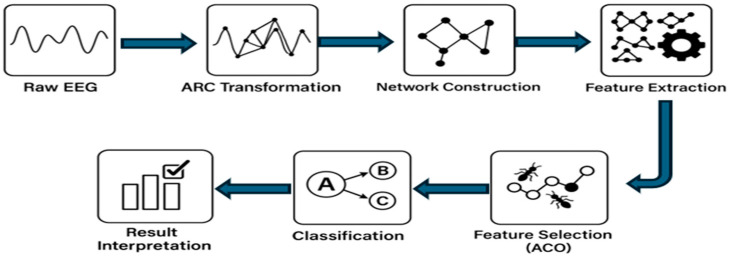
Systematic representation of the CONECT framework pipeline: EEG time series are converted into weighted complex networks using the ARC rule, followed by feature extraction, feature selection, and classification.

An overview of the CONECT pipeline is illustrated in Figure 1, and each stage is discussed in detail in the following subsections.

### 2.1. Dataset Description

This study uses the online benchmark dataset (i.e., OpenNeuro ds004504), which was acquired by an experienced team of neurologists from the 2nd Department of Neurology of AHEPA General Hospital of Thessaloniki [29]. The dataset contains EEG recordings of 88 subjects in a resting state with closed eyes using a referential Cz montage. The dataset comprises three categories:

1. Alzheimer’s disease (AD): 36 participants were diagnosed with AD group
2. Frontotemporal Dementia (FTD): 23 participants were diagnosed with FTD group
3. Healthy Subjects (CN): 29 participants were neurologically healthy subjects

To record the EEG signals, a Nihon Kohden 2100 clinical EEG system is used with 19 scalp electrodes (Fp1–O2) placed according to the 10–20 international system. For impedance checks and referencing, A1–A2 mastoid electrodes were used. The sampling rate was 500 Hz, with a resolution of 10 µV/mm. Each EEG time series in ds004504 is segmented into fixed-length epochs, each containing 20,000 time points, enabling fine-grained temporal analysis. The complete information about the dataset is available on the OpenNeuro repository [30]. This research study is conducted using the unprocessed EEG recordings (sub-0XX), rather than the preprocessed EEG ds004504 dementia data.

### 2.2. CONECT (Complex Network Conversion and Topology) Construction for EEG Data via the Angle-Relation Connection (ARC) Rule

This section discusses a novel geometric principle and the CONECT framework in detail. The workflow also covers the systematic transformation of each EEG segment into a weighted complex network using the novel Angle-Relation Connection (ARC) Rule. In this process, each time point in the EEG signal is modeled as a node, and edges are established based on an angular monotonicity condition: two points are connected only if the angular slope between them exceeds that of all intermediate points. This rule encodes signal curvature and directional change rather than relying on conventional line-of-sight or threshold-based comparisons.

Once edges are defined, the strength (weight) of each connection is determined using a composite function that incorporates amplitude difference, temporal separation, and angular orientation. This results in a richly structured weighted network, where both the presence and strength of edges reflect dynamic characteristics of the original EEG waveform.

#### 2.2.1. Angle-Relation Connection (ARC) Rule for Edge Formation

For a given EEG time series with amplitude values $x_{t}$, an edge is formed between nodes i and j, (j>i), if the following angular monotonicity condition holds for all intermediate indices k∈(i+1,j−1):

(1) $$\theta_{ik}<\theta_{ij}\;\mathrm{where}\;\theta_{ab}={tan}^{-1}\left(\frac{x_{b}-x_{a}}{b-a}\right),$$

This criterion ensures that intermediate points do not exhibit steeper angular slopes than the terminal pair, allowing the resulting network to capture directional geometric trends in the signal.

**Figure 2:**
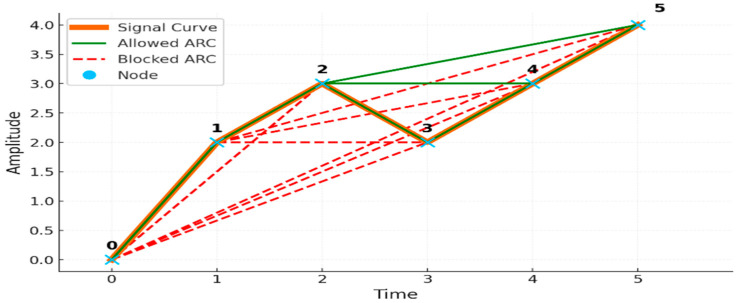
Illustrative example of the Angle-Relation Connection (ARC) Rule applied to a segment of EEG time series. The figure demonstrates how nodes (time points) are connected as edges based on the angular monotonicity condition of the ARC rule, capturing the geometric structure of the signal.

This criterion ensures that connections reflect the geometric structure and angular relationships inherent in the EEG signal. To better understand the network construction process, see Figure 2 for an illustrative example of the ARC Rule applied to a sample EEG segment.

The angular monotonicity condition, $\theta_{ik}<\theta_{ij}$, is designed to capture the moment-to-moment geometric stability of the EEG signal. A healthy EEG tends to show smooth angle transitions reflecting intact neural synchrony and stable oscillatory dynamics. Dementia disrupts these properties, producing sharper deflections, inconsistent curvature, and irregular phase progression. When these abnormalities occur, the natural monotonic relation between successive angular directions breaks down, resulting in more violations of $\theta_{ik}<\theta_{ij}$. Thus, this condition serves as a geometry-aware marker of temporal disorganization and reduced neural integrity, which are well-documented characteristics of dementia EEG.

#### 2.2.2. Edge Weight

The weight among the edges between two nodes, $\left(i,j\right)$, is computed by using the following equation:

(2) $$w_{ij}=\alpha \left|x_{j}-x_{i}\right|+\beta \frac{1}{\left|j-i\right|}+\gamma arctan\left(\frac{x_{j}-x_{i}}{j-i}\right),$$

where α, β, and γ are empirically determined parameters (e.g., α = 0.5, β = 0.3, γ = 0.2) that control the influence of amplitude difference, temporal separation, and angular orientation, respectively. The edge weight is tunable and directional edge-weighting function. The α–β–γ parameters in the proposed weighting function were selected through a combination of theoretical reasoning and preliminary development-phase tests performed on small representative EEG subsets. These early explorations showed that an α-dominant configuration best captured the curvature irregularities typically observed in dementia EEG, while appropriately scaled β and γ values preserved slope and amplitude dynamics without overwhelming curvature. To avoid arbitrary parameter choice in the α–β–γ weighting function, a small-scale exploratory analysis was conducted during the development phase. Several combinations of α (0.3–0.7), β (0.1–0.4), and γ (0.1–0.3) were tested on representative EEG segments to determine stable and meaningful contributions of each term in the weight expression.

The results indicated that α values below 0.4 under-emphasized clinically relevant amplitude differences, while values above 0.6 amplified noise; α = 0.5 provided a balanced influence. The β term controls the penalty for temporal separation: β < 0.15 made this effect negligible, whereas β > 0.35 biased the graph toward excessively local edges; β = 0.3 produced consistent temporal behavior. The γ term scales the slope-based angular component; γ < 0.15 reduced sensitivity to directional irregularities, while γ > 0.25 overly magnified angle fluctuations. A value of γ = 0.2 offered stable angular weighting. Based on these empirical observations, the combination (α = 0.5, β = 0.3, γ = 0.2) was selected as it yielded the most stable graph structures and the strongest preliminary classification performance, and was therefore used for all subsequent experiments.

Once the edge weights are computed, a CONECT graph is constructed that represents the relationships between EEG time series points.

**Figure 3:**
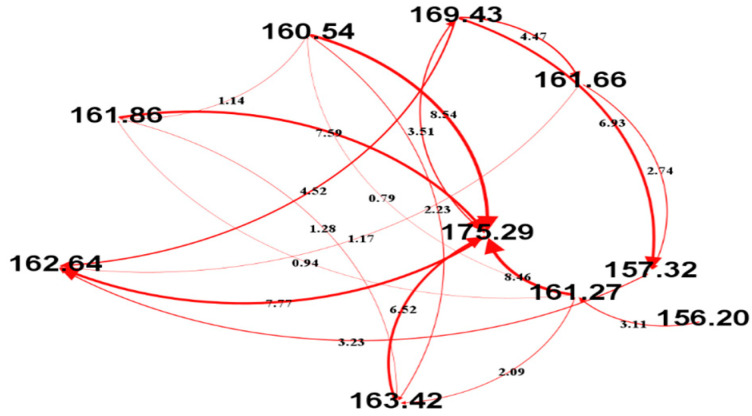
A CONECT graph constructed from 10 consecutive EEG values using the ARC rule. Node labels indicate the EEG values. Edge thickness and numerical labels represent computed weights. Arrow direction denotes the source-to-target node relationship.

To illustrate the weighted CONECT graph, we selected 10 data points [156.20, 161.28, 160.55, 161.87, 163.43, 175.29, 169.43, 161.67, 157.32, 162.65] from the real EEG data. Then, the ARC rule is applied to this authentic data segment/points to obtain the directed edges. The weight of each edge is calculated using Equation (2). The resulting CONECT graph is visualized in Figure 3, where node labels correspond to EEG data values and edge thickness/labels indicate computed weights. This concrete example demonstrates how both the connection logic and the weighting scheme transform raw EEG signals into a structured, interpretable network.

Unlike classical Visibility Graph (VG) and Horizontal Visibility Graph (HVG) methods, which construct edges based solely on amplitude-based visibility between points, the ARC rule explicitly models the geometric directionality and local angular smoothness of the EEG trajectory. VG/HVG capture peaks and troughs effectively but are insensitive to micro-scale angular variability, which is a known hallmark of dementia-related EEG disorganization. In contrast, the ARC rule uses an angular monotonicity criterion that responds directly to curvature instability and irregular directional transitions. Therefore, ARC offers improved sensitivity to the subtle temporal distortions observed in AD and FTD EEG signals.

Although deep-learning models such as CNNs, RNNs, Transformers, and graph neural networks are increasingly applied to EEG analysis, their advantages depend strongly on the structural properties of the data. Dementia EEG does not exhibit the dense spatial structure or large-scale redundancy typically required for effective deep representation learning. Moreover, deep models are prone to memorizing subject-specific patterns in subtle clinical EEG, require substantial computational resources, and offer limited interpretability; factors that restrict their clinical adoption. In contrast, the CONECT framework provides a geometry-aware and computationally efficient solution that captures physiologically meaningful patterns through ARC-based networks and interpretable geometric descriptors. As such, CONECT offers a more practical and transparent alternative to deep neural architectures for dementia classification.

### 2.3. Feature Extraction

Feature extraction plays a decisive role in a weighted complex network for interpreting the raw connectivity patterns of EEG signals into meaningful quantitative descriptors. It enables the discovery of topological, geometric, and statistical properties that characterize the underlying important dynamics of the EEG signal. The EEG graph features capture both local and global network organization by emphasizing the node importance and edge strength. Efficient feature extraction results in more accurate and robust classification outcomes by enhancing the interpretability and discriminative power of the model [31].

In this research study, we extracted a total of 15 features from the CONECT graph, including two proposed novel features and 13 baseline features used from prior literature.

#### 2.3.1. Proposed Novel Features

In this study, we proposed two novel graph features, the Weighted Angular Irregularity Index (WAII) and the Curvature-Based Edge Feature Index (CBEFI), that provide valuable insights into both topological complexity and temporal geometry of brain EEG activity. These features will enhance the EEG classification performance by introducing nonlinear, local, and geometry-aware characterization of network dynamics.

WAII will capture or highlight the local irregularity in the functional connectivity of the brain region, which is a common characteristic of dementia EEG. High values of WAII will reveal abnormal or unstable regional brain dynamics (such as AD).

CBEFI will capture the subtle changes in signal flow dynamics by modeling the geometric distortion of EEG signal propagation across channels.

1. Weighted Angular Irregularity Index (WAII):

WAII measures the normalized irregularity of outgoing edge weights for each node, thereby quantifying how much the strength of connections varies locally throughout the network. Network WAII is calculated as follows:

(3) $$WAII=\frac{1}{N}\sum_{i=1}^{N}\left\{\begin{array}{ll} \frac{std\left(S_{i}\right)}{mean\left(S_{i}\right)} & \mathrm{if}\;\left|S_{i}\right|>1\\ 0 & \mathrm{otherwise} \end{array}\right.,$$

2. Curvature-Based Edge Feature Index (CBEFI):

CBEFI quantifies the average local curvature along the edges of the network, reflecting sharpness or smoothness in the underlying EEG signal’s geometry. For each edge $\left(i,j\right)$, the local curvature is as follows:

(4) $$\kappa_{ij}=\left|\frac{x_{j}-2x_{k}+x_{i}}{{\left(t_{j}-t_{i}\right)}^{2}}\right|,$$

where k is the midpoint ($k=\frac{i+j}{2}$). The network CBEFI is as follows:

(5) $$CBEFI=\frac{1}{\left|E\right|}\sum_{\left(i,j\right)\in E}\kappa_{ij},$$

The Weighted Angular Irregularity Index (WAII) quantifies localized angular distortions in EEG trajectories, which are associated with neuronal disorganization and loss of temporal coordination commonly observed in dementia. Similarly, the Curvature-Based Edge Feature Index (CBEFI) captures curvature deviations in EEG signal geometry, reflecting changes in cortical connectivity and structural degradation associated with neurodegenerative progression. Prior studies have shown that EEG irregularity, geometric deformation, and fractal-based alterations are strong neurophysiological markers in Alzheimer’s disease and dementia, supporting the biological relevance of our proposed features. These findings align with evidence reported by Vicchietti et al. [32] and Yoder et al. [33], who demonstrated that geometric complexity and irregularity in EEG signals significantly correlate with dementia severity.

#### 2.3.2. Baseline Features

1. Edge Count:

This indicates network density and overall connectivity, reflecting brain network integration or potential loss in pathology [34]. The total number of edges in the network is as follows:

(6) $$C=\left|E\right|,$$

where E is the set of edges.

2. Edge Weight Kurtosis:

This highlights outlier connections and heavy-tailed distributions, revealing abnormal connectivity patterns [35]. The Kurtosis of edge weight distribution is as follows:

(7) $$K=\frac{1}{E}\sum_{e}\frac{{\left(w_{e}-\mu_{w}\right)}^{4}}{\sigma_{w}^{4}}-3,$$

where $w_{e}$ denotes the edge weights, $\mu_{w}$ is the mean, $\sigma_{w}$ is the standard deviation of the edge weights.

3. Edge Weight Mean:

This measures the average connection strength; lower values can indicate reduced brain synchronization [35]. The means of all edge weights are as follows:

(8) $$\mu_{w}=\frac{1}{\left|E\right|}\sum_{e=1}^{\left|E\right|}w_{e,}$$

4. Edge Weight Skewness:

This identifies asymmetry; a positive skew suggests a few dominant strong connections [35]. The skewness of the edge weight distribution is as follows:

(9) $$S=\frac{1}{E}\sum_{e}\frac{{\left(w_{e}-\mu_{w}\right)}^{3}}{\sigma_{w}^{3}},$$

5. Edge Weight Variance:

This captures the heterogeneity of connection strengths; a higher variance signals greater variability in connectivity [35]. The variance of the edge weights is as follows:

(10) $$\sigma_{w}^{2}=\frac{1}{E}\sum_{e}{\left(w_{e}-\mu_{w}\right)}^{2},$$

6. Gini Index:

Higher Gini values indicate a few connections dominate, mirroring inequality in network organization [36]. A measure of inequality in the distribution of edge weights is as follows:

(11) $$G=\frac{\sum_{i=1}^{n}\sum_{j=1}^{n}\left|x_{i}-x_{j}\right|}{2n^{2}\mu },$$

7. Global Efficiency:

This quantifies network integration, which is reduced in MCI/AD networks [37]. The average inverse shortest path length is as follows:

(12) $$E_{glob}=\frac{1}{N\left(N-1\right)}\sum_{i\neq j}\frac{1}{d_{ij}}\;,$$

where $d_{ij}$ is the shortest path length between i and j.

8. Modularity:

A high modularity reflects segregated subnetworks, often altered in disease [37]. The degree to which the network divides into communities is as follows:

(13) $$Q=\frac{1}{2m}\sum_{i,j}\left(A_{ij}-\frac{k_{i}k_{j}}{2m}\right)\delta \left(c_{i},c_{j}\right),$$

where $A_{ij}$ is the adjacency matrix, $k_{i}$ is the degree, m is the total edge weight, $c_{i}$ the community assignment.

9. Weighted Average Degree:

This represents the mean connectivity strength, sensitive to global changes in the network [37]. The average node strength (sum of edge weights per node) is as follows:

(14) $$\left\langle k^{w}\right\rangle =\frac{1}{N}\sum_{i=1}^{N}\sum_{j}w_{ij},$$

10. Average Degree:

This reflects network density, which is lower in pathological conditions [37]. The mean number of edges per node is as follows:

(15) $$\left\langle k\right\rangle =\frac{2\left|E\right|}{N},$$

11. Average Betweenness Centrality:

This measures the centrality of nodes in communication; reduced values may indicate network fragmentation [37]. The mean node betweenness centrality is as follows:

(16) $$\bar{BC}=\frac{1}{N}\sum_{i=1}^{N}BC_{i},$$

where $BC_{i}$ is the fraction of shortest paths passing through node i.

12. Average Node Strength:

This indicates the typical strength of network connections for each node [37]. The mean total weight per node is as follows:

(17) $$\left\langle s\right\rangle =\frac{1}{N}\sum_{i=1}^{N}\sum_{j}w_{ij},$$

13. Average Shortest Path Length:

A shorter path length indicates greater network integration; it is typically increased in disease [37]. The mean shortest path between all node pairs is as follows:

(18) $$L=\frac{1}{N\left(N-1\right)}\sum_{i\neq j}d_{ij},$$

where $d_{ij}$ is the shortest path length between i and j.

### 2.4. Feature Selection

The extraction of a comprehensive set of network features from the EEG graph is vital for capturing the subtle complexity of the signal and for differentiating between diagnostic groups. However, in EEG graph-based signals, using all available features simultaneously may introduce redundancy, increase the risk of overfitting, and obscure the most meaningful patterns. Therefore, effective feature selection is essential to reduce dimensionality, improve interpretability, and enhance model generalizability by identifying the subset of features that separate Healthy Controls from the clinical group (FTD, AD).

In this research study, Ant Colony Optimization (ACO) is employed as a bio-inspired optimization feature selection method for selecting the most informative features from the CONECT EEG graph. To the best of our knowledge, this is the first study to apply ACO for feature selection in dementia detection.

ACO is a metaheuristic algorithm inspired by the bio-inspired nature of ants that mimics how they collaborate to discover the shortest route to a food source by leaving and tracking pheromone trails. ACO employs machine learning to select a subset of features from a larger feature space to quickly determine which features are most informative.

ACO Process Overview: In each iteration, a population of artificial “ants” builds candidate feature subsets that are then evaluated via a classification model. The classification accuracy attained for each subset is used as its fitness score. Over successive iterations, the pheromone values are updated to strengthen feature combinations that attain the highest accuracy, enabling the algorithm to progressively converge toward the optimal subset, analogous to ants strengthening their most effective foraging paths [38].

In the paper, we implemented 30 ants per iteration and 50 total iterations, with each ant selecting a candidate subset of four to eight features per run. These parameter values were selected empirically to balance computational efficiency and stability of selected features, in accordance with established practices in the literature [38,39,40].

A brief sensitivity exploration was performed during the development phase to avoid arbitrary parameter selection. Testing different numbers of ants (20/30/40), iterations (30/50/70), and feature-subset ranges (3–6, 4–8, and 5–10) on smaller data splits showed clear performance trends. Configurations with too few ants or iterations typically led to premature convergence, while very large ranges increased redundancy without improving classification. The combination of 30 ants, 50 iterations, and a 4–8 feature window consistently produced stable convergence and favorable accuracy–runtime balance. Therefore, the final parameters were selected based on empirical stability rather than subjective choice.

### 2.5. Classification

The efficiency of a framework is assessed by its classification performance, which reflects its ability to generalize and accurately distinguish distinct neurodegenerative conditions from EEG patterns. In this context, the classification describes the task of automatically assigning each EEG recording to a diagnostic category (e.g., AD, FTD, or Healthy Control) based on the extracted CONECT network features. An effective classification model contributes as a valuable decision-support tool by bridging the gap between data analytics and clinical insight in early diagnosis and patient stratification [41,42].

In this study, five different classifiers: Random Forest, AdaBoost, Bagged Trees, K-Nearest Neighbors (KNN), and Radial Basis Function Support Vector Machine (RBF-SVM), were used to examine the classification performance of the newly developed features and established features. As in this study, we use the raw OpenNeuro ds004504 dataset (without preprocessing); therefore, it is essential to use multiple classifiers to handle the noise and variability in this raw data. Random Forest and Bagged Trees are ensemble methods and generate strong performance on complex and nonlinear data, as they provide robustness against noise and outliers by averaging or combining the decisions from multiple trees [43]. AdaBoost adaptively emphasized misclassified samples or difficult-to-classify data samples. It performs well on the noisy or imbalanced datasets by improving accuracy via combining weak learners into a strong predictive model [44]. KNN is a simple and non-parametric classifier that leverages local data similarities to detect subtle patterns in the feature space without assuming linear separability [45]. The RBF-SVM excels in modeling the nonlinear relationships present in the unfiltered brain dynamics [46]. Together, these classifiers ensured a comprehensive evaluation of the CONECT framework’s robustness and reliability when working with raw EEG data.

### 2.6. Performance Evaluation of CONECT Framework

To evaluate the performance of the proposed CONECT framework, we deployed multiple statistical metrics such as Accuracy, Sensitivity, Specificity, Precision, and F1-score. Each metric assessed the framework’s ability to correctly distinguish between AD, FTD, and HC while minimizing false classifications. The mathematical evaluation of all of these metrics is as follows [47]:

(19) $$Precision=\frac{TP}{TP+FP}$$

(20) $$Sensitivity=\frac{TP}{TP+FN}$$

(21) $$Accuracy=\frac{TP+TN}{TP+TN+FP+FN}$$

(22) $$Specificity=\frac{TN}{TN+FP}$$

(23) $$F1\;Score=2\;*\;\frac{Precision\;*\;Sensitivity}{Precision+Sensitivity}$$

where the positive class is AD, and the negative class is FTD (or Healthy Control):

- TP (True Positive): EEG from an AD patient correctly predicted as AD.
- FP (False Positive): EEG from an FTD or Healthy Control incorrectly predicted as AD.
- TN (True Negative): EEG from an FTD or Healthy Control correctly predicted as not AD.
- FN (False Negative): EEG from an AD patient incorrectly predicted as not AD.

## 3. Results and Discussion

The performance of the CONECT framework is evaluated on the OpenNeuro ds004504 EEG dataset. To reduce computational complexity while preserving essential signal characteristics, a fixed-length time window, equivalent to 40 s at a 500 Hz sampling rate, was extracted from each EEG channel for analysis. This window size was selected to balance the trade-off between data volume and temporal resolution while maintaining sufficient statistical power for downstream classification tasks.

To preserve the full temporal and spectral complexity of the EEG signals, we used the raw EEG data of the OpenNeuro ds004504 dataset for our analysis. For the analysis, the dataset is structured into the following classification task:

1. Binary classification Task
  - Alzheimer’s Disease (AD) vs. Healthy Controls (HC)
  - Alzheimer’s Disease (AD) vs. Frontotemporal Dementia (FTD)
  - Frontotemporal Dementia (FTD) vs. Healthy Controls (HC)
2. Multiclass classification
  - Alzheimer’s Disease (AD) vs. Healthy Controls (HC) vs. Frontotemporal Dementia (FTD)

Although the dataset contains 88 subjects, the number of recordings available for frontotemporal dementia (FTD) is comparatively limited. This imbalance is inherent to most public EEG datasets due to the lower clinical prevalence and scarcity of open-access FTD recordings. To reduce bias, all experiments used strict subject-level separation, cross-validated multiclass training, and within-fold ACO feature selection. Random Forest, which is robust to moderate imbalance, further contributed to stable performance across folds. Nevertheless, conclusions involving FTD should be interpreted with caution. Future work will expand the analysis to include additional FTD datasets or newly collected clinical EEG recordings to strengthen generalizability.

For classification performance of the CONECT framework, the data were divided into training and testing sets using a stratified 80/20 split, with shuffling enabled (random seed = 1) to ensure balanced class representation and reproducibility. The Random Forest (RF)classifier is used for multiclass classification with estimators = 250 trees. The choice of 250 trees is consistent with recommendations and established practice in biomedical classification literature [48]. The SVM model used in this study employed a Radial Basis Function (RBF) kernel to capture the nonlinear separability present in the CONECT-derived EEG features. The kernel parameters (C and γ) were selected through empirical tuning on a validation split to balance margin maximization and generalization. For the k-Nearest Neighbors classifier, several values of k (3, 5, 7, and 9) were explored, and k = 5 demonstrated the best trade-off between local sensitivity and noise robustness. These hyperparameter settings reflected the characteristics of the dataset and were used consistently throughout the experiments.

The complete list of extracted features and their corresponding indices is provided in Table 1. These indices reflect the exact order in which the features were processed during experimentation, beginning with 13 classical graph-theoretic measures, followed by the two novel geometry-informed metrics (WAII and CBEFI). This indexed ordering was preserved throughout the analysis to maintain consistency and comparability across all experimental settings.

### 3.1. Multiclass Classification Performance of the CONECT Framework

Table 2 highlights how combining new EEG-based features (WAII and CBEFI) with an optimization technique like ACO can make a real difference in accurately identifying dementia categories: Alzheimer’s (AD), Frontotemporal Dementia (FTD), and distinguishing them from healthy individuals. Table 2. Illustrates that initially with 13 baseline features (Edge Count, Edge Weight Kurtosis, Edge Weight Mean, Edge Weight Skewness, Edge Weight Variance, Gini Index, Global Efficiency, Modularity, Weighted Average Degree, Average Degree, Average Betweenness Centrality, Average Node Strength, Average Shortest Path Length), the model yields moderate accuracy of 70.96%. However, after adding two newly developed features (WAII + CBEFI), the classification accuracy increases to 81.14% without any optimization. Moreover, there is a significant increase in all other performance indicators, such as sensitivity, specificity, FI score, and precision. This clearly shows that our new proposed features are capturing aspects of the brain’s activity that standard features tend to overlook. The new features can also capture small irregularities or variations in signal geometry and connectivity that are typical in dementia cases and are often missed by conventional EEG features.

These classification performance improvements are further amplified through the application of Ant Colony Optimization (ACO) for feature selection. It is observed that deploying the ACO on 13 baseline features improved the accuracy to 79.34%. However, when ACO was applied to the combined 15-feature set (13 baseline features + WAII + CBEFI), the highest performance was achieved: 88.02% accuracy, 87.09% sensitivity, 93.71% specificity, 88.80% precision, and an F1-score of 87.72%. It is important to note that ACO identified a subset of 7 features (indices 1, 10, 14, 15, 9, 8, 7) among 15 feature indices. The results clearly demonstrate that the model’s performance can be improved with fewer, but more informative, inputs. And the selected features sustain the most discriminative information.

### 3.2. Performance Comparison of Feature-Selection Strategies: ACO vs. ReliefF and Boruta for Multiclass Classification

We conducted a comprehensive comparison of our Ant Colony Optimization (ACO) based feature-selection strategy with two widely used benchmark methods: ReliefF and Boruta. All methods were evaluated using the same classification model—i.e., Random Forest with 250 trees—to ensure methodological consistency and comparability. Table 3 summarizes the selected feature subsets and the resulting classification performance on the multiclass EEG dataset.

Both ReliefF and Boruta selected the top 10 features according to their respective importance scoring mechanisms, and each achieved an identical classification accuracy of 85.67%. In contrast, our ACO guided search produced a more compact and discriminative subset consisting of only seven features (Feature_1, Feature_7, Feature_8, Feature_9, Feature_10, Feature_14, Feature_15). Despite using fewer features, the ACO-selected subset yielded a superior accuracy of 88.02%, clearly outperforming the two benchmark approaches.

These findings demonstrate that the ACO mechanism effectively explores the feature space and identifies synergistic feature combinations that are not captured by traditional filter or wrapper-based methods. Notably, across all three feature-selection techniques, Feature_14 and Feature_15, our newly developed EEG-based features consistently emerged as the top-ranked contributors. Their repeated selection by ReliefF, Boruta, and ACO confirms their strong discriminative capability and validates the methodological significance of the proposed feature engineering strategy.

### 3.3. Binary-Class Classification Performance of the CONECT Framework

To gain deeper insights and a more granular assessment of the classification performance of the two newly proposed features and the CONECT framework, with and without ACO, we further investigate a series of binary classification analyses across each pairwise class combination (AD vs. HC, AD vs. FTD, and FTD vs. HC) using a Random Forest classifier. Table 4 Shows that without applying ACO, the Baseline 13 Features yield modest performance, such as in AD vs. HC, the accuracy was about 77.73%, which improved slightly on the addition of WAII and CBEFI—i.e., 83.81. However, after applying ACO, the model reached 93.52% accuracy on all 15 features. Similar enhancements in the classification performance were observed in AD vs. FTD (accuracy increased from 78.57% to 93.30%) and FTD vs. HC (from 91.37% to 97.46%), reflecting the generalizability and robustness of the optimized feature subsets (using the eight selected features).

These findings suggest that classical graph-based features alone may not fully capture the nuanced differences in EEG patterns among dementia subtypes. The inclusion of WAII and CBEFI captured irregularities and curvature-based dynamics more effectively, while ACO successfully eliminated redundant features, improving both accuracy and generalization. The Random Forest classifier, when supported by this enhanced feature pipeline, showed robust, stable, and highly reliable performance across all binary scenarios.

### 3.4. Comparative Evaluation of CONECT Framework for Dementia Classification Using Five Machine Learning Models

To evaluate the robustness of the proposed feature framework, five different classifiers were tested across both multiclass and binary classification tasks. The classifiers included Random Forest, AdaBoost, Bagged Trees, *k*-Nearest Neighbors (*k*-NN), and Radial Basis Function Support Vector Machine (RBF-SVM), each trained on a reduced set of highly informative graph-based features selected through ACO.

Table 5 illustrates that, in the multiclass scenario (AD vs. FTD vs. HC), the Random Forest classifier achieved the highest overall performance, with an accuracy of 88.0%, sensitivity of 87.1%, and specificity of 93.7%, using only seven features. Bagged Trees and *k*-NN (*k* = 5) followed with accuracies of 83.8% and 80.5%, respectively. AdaBoost showed moderate performance (78.1%), while RBF-SVM yielded the lowest accuracy (67.4%), indicating its limited generalization in this multiclass setup. Notably, Random Forest not only outperformed others in accuracy but also exhibited a strong F1-score (87.7%), confirming its stability across imbalanced class distributions.

In the AD vs. HC binary classification, all models improved substantially. Random Forest again outperformed other classifiers, achieving 93.5% accuracy, 94.3% precision, and a high F1-score of 93.4%, using only eight features. AdaBoost and *k*-NN (*k* = 5) also delivered competitive results (92.7% and 90.7% accuracy, respectively), while RBF-SVM lagged slightly at 87.9% accuracy. These results reinforce the discriminative capacity of the selected features in distinguishing Alzheimer’s Disease from healthy controls.

For the AD vs. FTD task, Bagged Trees marginally surpassed Random Forest, attaining the highest accuracy (94.6%) and F1-score (94.4%), followed closely by Random Forest with 93.3% accuracy. AdaBoost and *k*-NN (*k* = 5) delivered moderately strong results, while RBF-SVM again showed comparatively weaker performance (83.9% accuracy). The increased separability between AD and FTD reflects the effectiveness of the feature design in capturing subtle graph-based differences in disease phenotypes.

In the FTD vs. HC classification, Random Forest reached its peak performance, achieving 97.5% accuracy, 97.8% precision, and an F1-score of 97.4%. All classifiers performed well in this task, with AdaBoost, Bagged Trees, and *k*-NN (*k* = 5) all exceeding 93% accuracy. RBF-SVM, while still slightly behind, yielded 91.9% accuracy, its best result across all binary tasks. These findings suggest that FTD and HC are highly distinguishable when leveraging optimized graph features, especially with ensemble-based classifiers.

Across both multiclass and binary evaluations, Random Forest consistently demonstrated superior performance, particularly when paired with the reduced feature sets derived from ACO and the proposed WAII and CBEFI features. Ensemble methods like Bagged Trees and AdaBoost also performed robustly, especially in binary tasks. The lower accuracy of RBF-SVM across most comparisons highlights its limitations in handling this feature representation. Overall, these results validate the reliability and discriminative power of the CONECT framework across diverse classification settings.

Random Forest achieved the highest multiclass accuracy because its algorithmic characteristics closely match the structure of the EEG-derived features. The CONECT–ARC framework produces heterogeneous and nonlinearly distributed geometric features, including slope-based angular information, amplitude irregularity measures, and distance-dependent weights. Random Forest can naturally model complex, multi-modal decision boundaries through its ensemble of decorrelated trees, while also capturing higher-order feature interactions that arise from ACO-selected feature subsets. The built-in averaging mechanism also reduces variance, making it particularly suitable for noisy biomedical data such as EEG. In contrast, RBF-SVM performed comparatively worse because a single global RBF kernel struggled to represent the highly nonlinear and partially overlapping distributions of AD, FTD, and HC in the feature space. Moreover, multiclass SVM relies on multiple pairwise boundaries, which become sensitive to feature imbalance and class overlap. The RBF parameters (C and γ) were tuned empirically but were not exhaustively optimized due to computational cost, likely contributing to reduced performance. These intrinsic differences explain the superior performance of Random Forest in the multiclass dementia-classification task.

### 3.5. Feature Set Performance Summary

Table 6 presents the ablation study evaluating the incremental contribution of WAII, CBEFI, and ACO-based feature selection. Starting from the baseline model with 13 graph features, each additional module leads to consistent performance improvements across all binary and multiclass tasks. WAII and CBEFI individually enhance discrimination ability, and their combination further strengthens the feature representation. The Full CONECT configuration, which integrates both new geometric features and ACO-driven feature selection, achieves the highest accuracy across all evaluations. The ablation results clearly demonstrate that each proposed component independently improves accuracy, and that their combination leads to substantial performance gains. Specifically, adding WAII improved the multiclass accuracy from 70.96% to 77.31%, while adding CBEFI resulted in 77.01%. When both WAII and CBEFI were used together, the accuracy increased further to 81.14%. Incorporating the ACO-based feature-selection mechanism (Full CONECT) yielded the highest performance across all metrics, achieving 93.30% (AD vs. FTD), 97.46% (FTD vs. HC), 93.52% (AD vs. HC), and 88.02% in the multiclass scenario.

These results validate the effectiveness of each enhanced module and collectively confirm that the full CONECT framework provides the strongest and most discriminative feature representation for dementia subtype classification.

### 3.6. Contribution of WAII and CBEFI to Feature Selection

To assess the individual contribution of each graph-derived feature within the CONECT framework, a detailed analysis of feature selection frequency was conducted using the Ant Colony Optimization (ACO) algorithm over 50 independent runs with 30 agents. The objective was to observe which features consistently emerged as part of the optimal subset for classification tasks, reflecting their importance and generalizability across multiple data splits.

**Figure 4:**
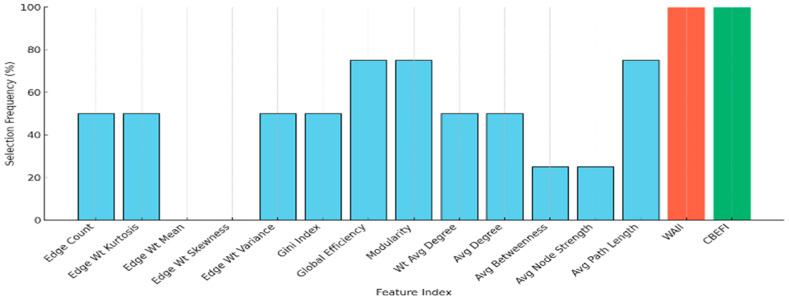
Feature selection frequency (%) across all four classification tasks based on final ACO-optimized subsets. The newly developed geometric features (WAII and CBEFI) were selected for 100% of tasks, indicating their consistent importance in distinguishing dementia subtypes.

As shown in Figure 4, the two newly developed features, WAII (Weighted Angular Irregularity Index) and CBEFI (Curvature-Based Edge Feature Index), have achieved a 100% selection frequency, meaning they were included in every optimal feature subset across all iterations and classification tasks. This consistent prioritization strongly indicates their robust discriminative ability, outperforming traditional metrics such as edge count, node degree, clustering-based metrics, and even widely used global graph measures like modularity and global efficiency.

The high selection rate of WAII and CBEFI validates their ability to capture nuanced variations in EEG network topology that are often overlooked by classical descriptors. In contrast, several conventional features, such as average nodal strength and average path length, were selected in less than 30% of cases, suggesting limited standalone relevance in dementia classification.

Furthermore, this trend was independently corroborated by Random Forest feature importance rankings, where WAII and CBEFI consistently ranked among the top five contributors across binary and multiclass tasks. Together, these findings not only support the inclusion of these novel features but also demonstrate how biologically informed, structure-aware graph metrics can enhance classification outcomes in neurodegenerative disease detection.

### 3.7. Cross-Validation Performance Evaluation

**Figure 5:**
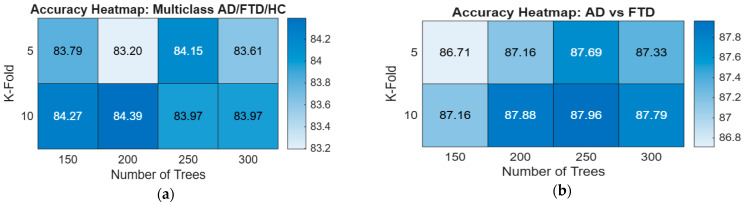

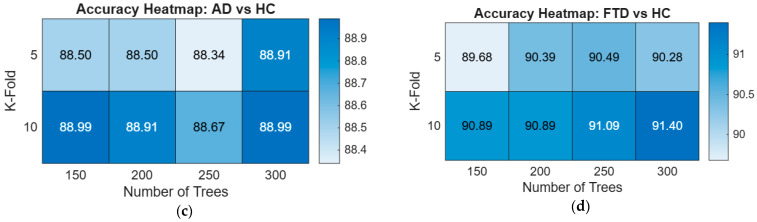
Accuracy heatmaps for Random Forest classification performance across four experimental settings: (**a**) Multiclass AD/FTD/HC, (**b**) AD vs. FTD, (**c**) AD vs. HC, and (**d**) FTD vs. HC. Each heatmap illustrates the effect of varying the number of trees (150, 200, 250, 300) and cross-validation folds (5-fold and 10-fold) on accuracy.

Figure 5 presents four heatmaps summarizing the impact of Random Forest hyperparameters: number of trees (150–300) and K-fold values (5-fold and 10-fold), across all classification tasks. The multiclass AD/FTD/HC experiment showed stable performance (83.20–84.39%), with the highest accuracy observed at 200 trees using 10-fold CV. In the binary classifications, accuracy consistently improved with 10-fold CV, reflecting enhanced robustness. For AD vs. FTD, the best performance (87.96%) occurred at 250 trees with 10-fold CV, while AD vs. HC produced uniformly strong results (88.34–88.99%).

The FTD vs. HC task demonstrated the highest overall accuracy, reaching 91.40% at 300 trees using 10-fold CV, indicating a clear separability between these classes. Overall, the heatmaps show that 10-fold cross-validation yields more reliable and slightly higher accuracies, and that increasing the number of trees improves performance until convergence around 250–300 trees.

### 3.8. Comparison with Recent Studies Across Dementia Classification Tasks

Table 7 summarizes recent EEG-based dementia classification studies, many of which report high accuracies but often rely on extensive preprocessing pipelines involving heavy filtering, artifact rejection, and selective epoching. While these steps improve signal quality, they also reduce clinical applicability and may inflate performance. In contrast, the CONECT framework was developed and evaluated entirely on raw or unprocessed EEG data from the OpenNeuro ds004504 dataset, without applying filtering, ICA, or manual artifact removal, and bringing the analysis closer to real-world clinical conditions. CONECT achieved classification performance that matches or exceeds that of prior studies. This result demonstrates the framework’s potential for reliable, real-world application without reliance on heavy data cleaning, thus offering both technical innovation and immediate translational value. Despite using an unfiltered EEG, CONECT outperformed or matched State-of-the-Art results, confirming its robustness, practical relevance, and potential for real-time, scalable dementia screening.

The geometry-aware weighting and ARC-based connectivity in CONECT inherently encode temporal curvature, directional change, and local amplitude dynamics of the EEG signal. As a result, the extracted network features reflect meaningful neurophysiological patterns: AD recordings typically show reduced integration and smoother curvature dynamics, consistent with global synaptic disconnection; FTD signals exhibit sharper local transitions and irregularity aligned with frontal–temporal degeneration; while HC recordings maintain richer geometric variability. Because CONECT features are computed per channel, these dynamics can be associated with brain-region-specific dysfunction, providing a foundation for future topographic and disease progression analyses.

### 3.9. Behavior of WAII and CBEFI Across Groups

The proposed WAII and CBEFI features directly encode geometry-aware properties of the EEG time series. WAII quantifies angular instability within the ARC-weighted network, highlighting rapid directional changes that reflect temporal irregularity. CBEFI measures curvature-based energy, capturing the deformation and smoothness of the signal trajectory. In our experiments, both features were repeatedly selected by the ACO search process and were instrumental in elevating classification performance, indicating that they encode stable and discriminative geometric patterns across HC, AD, and FTD. These patterns align with the mathematical properties of the features: AD recordings typically exhibit reduced curvature (lower CBEFI) and greater angular instability (higher WAII), while FTD tends to present stronger deformation in curvature profiles relative to AD. Thus, WAII and CBEFI provide meaningful geometry-based distinctions between the groups, further validating their role as novel EEG biomarkers.

### 3.10. Computational Efficiency

To evaluate the practical feasibility of the CONECT framework, we measured the computational cost of each processing stage on a standard workstation (Intel Core i7 CPU, 16 GB RAM, MATLAB R2024b). Constructing the ARC-based weighted network for a single EEG channel (20,000 samples) required approximately 20 s, which is expected given that the ARC rule performs angular monotonicity checks across numerous point pairs. Feature extraction from the resulting weighted network required an additional 2–3 s per channel. Classification using a trained Random Forest model executed almost instantaneously (<0.05 s). The ACO stage—due to 30 ants × 50 iterations—is more computationally demanding, but it is performed only once during training and is not required during deployment. Overall, processing all 19 channels of a single EEG recording required approximately 7–8 min, which remains practical for offline analysis. With channel-wise parallelization or GPU acceleration, the total runtime can be substantially reduced, making the framework suitable for accelerated or near-real-time clinical decision support in future applications.

## 4. Conclusions

This study presents a novel framework named CONECT for EEG-based dementia classification based on multiple technical innovations. Foremost is the development of the ARC rule, a new and original technique for constructing brain connectivity networks from EEG data with precise geometric criteria. The framework also introduced a tunable heuristic edge link that allows for dynamic adjustment of network weights based on the specific properties of the EEG signals and dataset, thereby enhancing adaptability and robustness. Two novel geometric features, the Weighted Angular Irregularity Index (WAII) and the Curvature-Based Edge Feature Index (CBEFI), were developed to quantify network irregularities and curvatures that are not captured by traditional graph metrics. To accurately identify the most discriminative feature subsets, the Ant Colony Optimization (ACO) method is used for feature selection. Consistently, WAII and CBEFI emerged as key features across all classification tasks, underscoring their value as novel EEG biomarkers feature for dementia. All analyses are performed on non-processed EEG from the OpenNeuro ds004504 dataset, with no further artifact correction or filtering. CONECT achieved State-of-the-Art accuracy in both multiclass and binary discrimination, matching or surpassing the State-of-the-Art that applied far more extensive preprocessing techniques. The transparent methodology and adaptability of CONECT make it well-suited for both research and clinical applications, especially in settings where aggressive data cleaning is impractical. The improved performance observed in the ablation study highlights the discriminative value of the newly proposed WAII and CBEFI features. WAII captures localized angular irregularities in EEG morphology, while CBEFI quantifies curvature-driven geometric deviations, both of which are highly sensitive to dementia-related neural degradation. By incorporating these geometry-aware biomarkers, the feature space becomes more expressive and better aligned with disease-specific signal dynamics, resulting in consistent accuracy gains across all classification tasks. The integration of a novel network construction approach, a tunable heuristic edge weight mechanism, innovative features, and metaheuristic optimization positions CONECT as a robust and generalizable approach for EEG-based neurodegenerative disease diagnosis. Future research will focus on additional validation across diverse datasets (e.g., EEG epileptic seizure, sleep apnea, or alcohol use disorder) and clinical settings, and on exploring additional applications for these network-based biomarkers.

## Figures and Tables

**Table 1:** Lists the complete sequence of extracted features and their respective categories, which are referenced by index in subsequent analyses.

Feature Index	Feature Name	Category
1	Edge Count	Classical
2	Edge Weight Kurtosis	Classical
3	Edge Weight Mean	Classical
4	Edge Weight Skewness	Classical
5	Edge Weight Variance	Classical
6	Gini Index	Classical
7	Global Efficiency	Classical
8	Modularity	Classical
9	Weighted Average Degree	Classical
10	Average Degree	Classical
11	Average Betweenness Centrality	Classical
12	Average Node Strength	Classical
13	Average Path Length	Classical
14	Weighted Angular Irregularity Index (WAII)	Novel
15	Curvature-Based Edge Feature Index (CBEFI)	Novel

**Table 2:** Random Forest-based multiclass classification performance of the CONECT framework for AD, FTD, and HC using Baseline and Proposed Novel Features with and without ACO.

Performance Metric	ACO (Applied)	Feature Set	Value (%)	Selected Indices
**Accuracy**	No	Baseline 13 Features	70.96	1–13
		Baseline 13 Features + (Proposed Novel Features)	81.14	1–15
	Yes	Baseline 13 Features	79.34	8, 7, 1, 13, 4, 10, 9, 6
		Baseline 13 Features + (Proposed Novel Features)	**88.02**	1, 10, 14, 15, 9, 8, 7
**Sensitivity**	No	Baseline 13 Features	70.10	1–13
		Baseline 13 Features + (Proposed Novel Features)	80.17	1–15
	Yes	Baseline 13 Features	78.36	8, 7, 1, 13, 4, 10, 9, 6
		Baseline 13 Features + (Proposed Novel Features)	**87.09**	1, 10, 14, 15, 9, 8, 7
**Specificity**	No	Baseline 13 Features	84.70	1–13
		Baseline 13 Features + (Proposed Novel Features)	90.02	1–15
	Yes	Baseline 13 Features	89.08	8, 7, 1, 13, 4, 10, 9, 6
		Baseline 13 Features + (Proposed Novel Features)	**93.71**	1, 10, 14, 15, 9, 8, 7
**Precision**	No	Baseline 13 Features	72.65	1–13
		Baseline 13 Features + (Proposed Novel Features)	82.59	1–15
	Yes	Baseline 13 Features	80.75	8, 7, 1, 13, 4, 10, 9, 6
		Baseline 13 Features + (Proposed Novel Features)	**88.80**	1, 10, 14, 15, 9, 8, 7
**F1-Score**	No	Baseline 13 Features	70.98	1–13
		Baseline 13 Features + (Proposed Novel Features)	81.04	1–15
	Yes	Baseline 13 Features	79.23	8, 7, 1, 13, 4, 10, 9, 6
		Baseline 13 Features + (Proposed Novel Features)	**87.72**	1, 10, 14, 15, 9, 8, 7

**Table 3:** Performance Analysis of ReliefF, Boruta, and ACO Feature Selection with Random Forest Classifier.

Feature Selection Method	Selected Features (Top 10 or Optimal)	No. of Selected Features	Classifier	Accuracy (%)
ReliefF	14, 15, 2, 4, 6, 8, 10, 1, 3, 5	10	Random Forest (250 trees)	85.67%
Boruta	14, 15, 6, 3, 2, 4, 13, 7, 1, 8	10	Random Forest (250 trees)	85.67%
Ant Colony Optimization (ACO)	14, 15, 1, 10, 9, 8, 7	7	Random Forest (250 trees)	88.02%

**Table 4:** Performance Evaluation of ACO and Proposed Graph Features (WAII and CBEFI) for Binary Classification under the CONECT Framework.

Comparison	ACO (Applied)	Feature Set	No. of Features	Accuracy (%)	Sensitivity (%)	Specificity (%)	Precision (%)	F1-Score (%)	Selected Indices
**AD vs. HC**	No	Baseline 13 Features	13	77.73	76.97	76.97	77.74	77.19	1–13
	No	All 15 (Baseline 13 Features + WAII + CBEFI)	15	83.81	83.07	83.07	84.13	83.39	1–15
	Yes	Baseline 13 Features	7	87.45	87.25	87.25	87.32	87.29	2, 8, 5, 3, 7, 13, 10
	Yes	All 15 (Baseline 13 Features + WAII + CBEFI)	8	**93.52**	**92.91**	**92.91**	**94.28**	**93.36**	13, 5, 7, 15, 14, 6, 9, 2
**AD vs. FTD**	No	Baseline 13 Features	13	78.57	77.03	77.03	77.53	77.25	1–13
	No	All 15 (Baseline 13 Features + WAII + CBEFI)	15	85.71	83.71	83.71	85.95	84.53	1–15
	Yes	Baseline 13 Features	7	90.18	88.20	88.20	91.30	89.31	9, 3, 5, 13, 1, 8, 7
	Yes	All 15 (Baseline 13 Features + WAII + CBEFI)	8	**93.30**	**92.08**	**92.08**	**93.96**	**92.84**	5, 6, 14, 8, 10, 15, 13, 2
**FTD vs. HC**	No	Baseline 13 Features	13	91.37	90.83	90.83	91.75	91.17	1–13
	No	All 15 (Baseline 13 Features + WAII + CBEFI)	15	92.39	92.22	92.22	92.33	92.27	1–15
	Yes	Baseline 13 Features	8	90.86	90.50	90.50	90.96	90.69	9, 8, 7, 10, 6, 2, 5, 12
	Yes	All 15 (Baseline 13 Features + WAII + CBEFI)	8	**97.46**	**97.13**	**97.13**	**97.83**	**97.41**	7, 15, 11, 1, 13, 14, 12, 8

**Table 5:** Performance Comparison of Five Machine Learning Models on Multiclass and Binary Dementia Classification Using ACO-Selected Graph Features under the CONECT Framework.

Task	Classifier	Accuracy (%)	Sensitivity (%)	Specificity (%)	Precision (%)	F1-Score (%)	Num Features
**Multiclass**	Random Forest	88.0	87.1	93.7	88.8	87.7	7
	AdaBoost	78.1	76.0	88.2	81.2	77.3	8
	Bagged Trees	83.8	83.6	91.6	84.0	83.8	7
	*k*-NN (*k* = 5)	80.5	79.7	89.8	81.2	80.3	8
	RBF SVM	67.4	66.4	82.9	68.5	67.1	8
**AD vs. HC**	Random Forest	93.5	92.9	92.9	94.3	93.4	8
	AdaBoost	92.7	92.5	92.5	92.7	92.6	7
	Bagged Trees	89.1	88.3	88.3	89.9	88.7	8
	*k*-NN (*k* = 5)	90.7	90.4	90.4	90.8	90.5	8
	RBF SVM	87.9	87.7	87.7	87.7	87.7	8
**AD vs. FTD**	Random Forest	93.3	92.1	92.1	94.0	92.8	8
	AdaBoost	90.2	88.8	88.8	90.4	89.5	7
	Bagged Trees	94.6	94.2	94.2	94.6	94.4	7
	*k*-NN (*k* = 5)	86.2	84.9	84.9	85.7	85.3	8
	RBF SVM	83.9	80.4	80.4	86.4	81.8	8
**FTD vs. HC**	Random Forest	97.5	97.1	97.1	97.8	97.4	8
	AdaBoost	94.9	95.0	95.0	94.8	94.9	8
	Bagged Trees	94.4	93.9	93.9	94.9	94.3	8
	*k*-NN (*k* = 5)	93.4	93.4	93.4	93.3	93.3	7
	RBF SVM	91.9	92.0	92.0	91.7	91.8	8

**Table 6:** Ablation study evaluating the comparison of binary and multiclass classification performance (Accuracy) using baseline and proposed feature configurations, including WAII, CBEFI, and ACO-based feature selection (Full CONECT).

Configuration	Features Used	AD vs. FTD (%)	FTD vs. HC (%)	AD vs. HC (%)	Multiclass (%)
Baseline	13 graph features	78.57	91.37	77.73	70.96
Baseline + WAII	14 features	81.70	88.89	84.21	77.31
Baseline + CBEFI	14 features	83.04	87.37	80.97	77.01
Baseline + WAII + CBEFI	15 features	85.71	92.39	83.81	81.14
Baseline + WAII + CBEFI + ACO (Full CONECT)	Selected features	93.30	97.46	93.52	88.02

**Table 7:** Comparative Performance of Recent EEG-Based Dementia Studies and the Proposed CONECT Framework.

Comparison Group	Author	Year	Accuracy (%)
**AD vs. HC**	Hasan, M.M. [49]	2024	76.0
	Ma et al. [50]	2024	76.9
	Zheng, H. et al. [51]	2024	87.7
	Chen, Y. et al. [52]	2023	87.3
	Lal, U. et al. [53]	2024	91.0
	Proposed CONECT		93.5
**FTD vs. HC**	Hasan, M.M. [49]	2024	77.0
	Zheng, H. et al. [51]	2024	82.7
	Ma et al. [50]	2024	90.4
	Chen, Y. et al. [52]	2023	82.9
	Lal, U. et al. [53]	2024	93.0
	Proposed CONECT		97.5
**AD vs. FTD**	Zheng, H. et al. [51]	2024	72.9
	Hasan, M.M. [49]	2024	78.0
	Chen, Y. et al. [52]	2023	81.6
	Lal, U. et al. [53]	2024	91.0
	Proposed CONECT		93.3
**Multiclass (AD vs. FTD vs. HC)**	Chen, Y. et al. [52]	2023	79.1
	Hasan, M.M. [49]	2024	82.0
	Proposed CONECT		88.0

## Data Availability

The data used in this research study are available at the following link: Available online: https://openneuro.org/datasets/ds004504/versions/1.0.8 (accessed on 15 October 2025).
